# The Long Pentraxin PTX3 as a New Biomarker and Pharmacological Target in Age-Related Macular Degeneration and Diabetic Retinopathy

**DOI:** 10.3389/fphar.2021.811344

**Published:** 2022-01-07

**Authors:** Matteo Stravalaci, Mariantonia Ferrara, Varun Pathak, Francesca Davi, Barbara Bottazzi, Alberto Mantovani, Reinhold J. Medina, Mario R. Romano, Antonio Inforzato

**Affiliations:** ^1^ IRCCS Humanitas Research Hospital, Rozzano, Italy; ^2^ Eye Center, Humanitas Gavazzeni-Castelli, Bergamo, Italy; ^3^ School of Medicine, Dentistry, and Biomedical Sciences, Wellcome-Wolfson Institute for Experimental Medicine, Queen’s University Belfast, Belfast, United Kingdom; ^4^ Department of Biomedical Sciences, Humanitas University, Rozzano, Italy; ^5^ The William Harvey Research Institute, Queen Mary University of London, London, United Kingdom

**Keywords:** age-related macular degeneration, diabetic retinopathy, complement, inflammation, PTX3

## Abstract

Age related macular degeneration (AMD) and diabetic retinopathy (DR) are multifactorial, neurodegenerative and inflammatory diseases of the eye primarily involving cellular and molecular components of the outer and inner blood-retina barriers (BRB), respectively. Largely contributed by genetic factors, particularly polymorphisms in complement genes, AMD is a paradigm of retinal immune dysregulation. DR, a major complication of diabetes mellitus, typically presents with increased vascular permeability and occlusion of the retinal vasculature that leads, in the proliferative form of the disease, to neovascularization, a pathogenic trait shared with advanced AMD. In spite of distinct etiology and clinical manifestations, both pathologies share common drivers, such as chronic inflammation, either of immune (in AMD) or metabolic (in DR) origin, which initiates and propagates degeneration of the neural retina, yet the underlying mechanisms are still unclear. As a soluble pattern recognition molecule with complement regulatory functions and a marker of vascular damage, long pentraxin 3 (PTX3) is emerging as a novel player in ocular homeostasis and a potential pharmacological target in neurodegenerative disorders of the retina. Physiologically present in the human eye and induced in inflammatory conditions, this protein is strategically positioned at the BRB interface, where it acts as a “molecular trap” for complement, and modulates inflammation both in homeostatic and pathological conditions. Here, we discuss current viewpoints on PTX3 and retinal diseases, with a focus on AMD and DR, the roles therein proposed for this pentraxin, and their implications for the development of new therapeutic strategies.

## Introduction

Diabetic retinopathy (DR) and age-related macular degeneration (AMD) are leading causes of vision loss in working-age and elderly individuals, respectively, in developed countries (Cheung et al., 2010; Wong et al., 2014). In spite of distinctive clinical presentations and pathogenetic mechanisms, both diseases are multifactorial and neurodegenerative, and have in chronic inflammation a common driver. Cellular and molecular components of the outer blood-retinal barrier (oBRB) in AMD and inner BRB (iBRB) in DR are primarily involved in these pathologies. AMD manifests itself with accumulation of retinal pigment epithelium (RPE) abnormalities and drusen, extracellular deposits located between the RPE and Bruch’s membrane (BrM) (Parmeggiani et al., 2013). On the other hand, DR is characterized by capillary occlusion, decreased retinal perfusion, microvascular destabilization, and increased vascular permeability, which collectively lead to retinal ischemia and vascular abnormalities (e.g., microaneurysms, retinal hemorrhages) (Semeraro et al., 2015). In both cases, a status of chronic and local inflammation sets in place that propels and sustains progression to advanced pathology. In this regard, exudative macular edema, neovascularization and hemorrhages, responsible for structural damage to the retina and fibrosis, are characteristic of DR and neovascular AMD (Govetto et al., 2020), whereas geographic atrophy, degeneration of the RPE and photoreceptors are typical processes of dry AMD (Parmeggiani et al., 2013). Interestingly, among the optical coherence tomography (OCT) biomarkers identified for these diseases (Ceravolo et al., 2020; Sitnilska et al., 2021), intraretinal hyperreflective foci in optical coherence tomography (OCT), representative of microglial activation and, thus, intraretinal inflammation, have been proposed as biomarkers of AMD and DR (Schreur et al., 2020; Sitnilska et al., 2021; Wu et al., 2021), indicating that local inflammatory reactions in the eye might have diagnostic potential (e.g., to monitor disease progression and/or response to therapy) in addition to play a direct role as pathogenetic mechanisms.

AMD is regarded as the paradigm of retinal immune dysregulation, in that local overactivation of the complement system (and the following inflammation) is a primary pathogenetic mechanism of AMD (Ambati et al., 2013; Clark and Bishop, 2018). In this regard, many factors contribute to the risk of disease, including age-related changes in structure/function of the RPE and BrM, oxidative stress, lifestyle, and, more importantly, genetics (Armento et al., 2021). Most of the polymorphisms associated with onset and progression of AMD map in or nearby genes of the complement system, particularly those of the alternative pathway (AP) (Fritsche et al., 2016). Of these, the Y402H polymorphism in the factor H gene (*CFH*) is the greatest (single) genetic risk factor of developing AMD (Hageman et al., 2005; Fritsche et al., 2016). This polymorphism affects the coding sequence of factor H (major soluble inhibitor of the AP) and its truncated form factor H-like protein 1 (FHL-1), and alters the binding of these proteins to sulfated glycosaminoglycans (GAGs) of the BrM (Clark et al., 2010). This extracellular matrix is devoid of any other inhibitor of the AP, therefore the restricted binding specificity of the pathological variant of factor H (and FHL-1) is believed to cause dysregulated complement activation (Clark and Bishop, 2018). This leads to generation of the anaphylatoxins C3a and C5a and the sub-lytic C5b-9 complexes, which induce the RPE to express inflammatory cytokines and growth factors (e.g., vascular endothelial growth factor, VEGF) (Lueck et al., 2011; Lueck et al., 2015). Complement fragments and RPE-derived factors cooperatively promote recruitment and activation of immune cells (Behnke et al., 2020; Ogura et al., 2020) that propagate the inflammatory reaction in the eye, eventually leading to damage and dysfunction of the oBRB (Natoli et al., 2017). In addition to inflammation, complement overactivation has been associated with impaired antioxidant potential and energy metabolism in the RPE cells (Sivapathasuntharam et al., 2019; Armento et al., 2020), and accumulation of retinal lipids in the drusen (Acar et al., 2020). Furthermore, age-related changes in the RPE and BrM, and smoking both contribute to complement activation, retinal inflammation and oxidative stress, pointing to a close interaction between diverse risk factors (Pietkiewicz et al., 2008; Wang et al., 2014; Woodell and Rohrer, 2014; Clark et al., 2017; Fields et al., 2017; Brown et al., 2019).

In DR, hyperglycemia promotes vascular dysfunction and neuroinflammation through multiple biochemical mechanisms, including activation of the polyol pathway, increased expression of cytokines and growth factors (e.g., tumor necrosis factor-alpha, TNF-α, pro-inflammatory interleukins, adiponectin, erythropoietin, VEGF, angiopoietin-2), oxidative stress, enhanced production of advanced glycation and lipoxidation end-products (AGE, ALE, respectively), hemodynamic changes, and leukostasis and oxidative stress (the latter also involved in AMD and other retinal pathologies) (Lorenzi, 2007; Costagliola et al., 2013; Semeraro et al., 2014; Semeraro et al., 2015; Khalaf et al., 2017; Toro et al., 2019; Kinuthia et al., 2020; Pietras-Baczewska et al., 2021). In particular, reactive oxygen species (ROS), AGE, ALE, and pro-inflammatory molecules collectively lead to activation of microglia, which is considered a major mechanism of neuroinflammation (Sharma et al., 2012; Semeraro et al., 2015; Subedi et al., 2020). Activated microglial cells in turn synthesize and release pro-inflammatory cytokines (e.g., TNF-α, IL-1β, and IL-6) and cytotoxic molecules (e.g., ROS and reactive nitrogen species, RNS) that propagate the local inflammatory response with endothelial damage, loss of pericytes, iBRB disruption, and vascular dysfunction (Scholz et al., 2015; Kinuthia et al., 2020). Neuroinflammation is further amplified by overactivation of the complement system and other glial cells, such as retinal astrocytes (Kinuthia et al., 2020; Shahulhameed et al., 2020). Moreover, breakdown of the iBRB favors the transfer of circulating pro-inflammatory factors, including chemokines, cytokines, and immune cells, into the inner retina which further promotes local immune dysregulation and retinal neurovascular damage, thus leading to DR progression (Kinuthia et al., 2020).

AMD and DR share cellular and molecular components of major inflammatory pathways, including the long pentraxin 3 (PTX3), long-known as a key mediator of vascular (Presta et al., 2018; Ristagno et al., 2019) and complement-dependent (Doni et al., 2019; Haapasalo and Meri, 2019) inflammation, and recently emerging as a novel player in retinal neurodegeneration (Wang et al., 2016; Stravalaci et al., 2020). Here, we revisit available clinical and preclinical evidence on the role(s) of PTX3 in AMD and DR, with major regard to current hypotheses on the involvement of this pentraxin in their pathogenesis, and its potential as a diagnostic/prognostic biomarker (see Table 1).

**TABLE 1:** Studies describing expression and localization of PTX3 in AMD and DR.

References	Source	Treatment	Localization	Main findings
An et al. (2008), Woo et al. (2013), Juel et al. (2015), Stravalaci et al. (2020)	ARPE-19 cell line	TNF-α^a^IL-1β	secreted	PTX3 was overexpressed by ARPE-19 cells in inflammatory conditions, and the released protein had complement-inhibiting properties
Yamada et al. (2008), Wang et al. (2016)	ARPE-19 cell line	ox-LDL 4-HNE	secreted	PTX3 expression was induced by oxidative stress
Hwang et al. (2019)	ARPE-19 cell line primary H-RPE cells	NaIO_3_	secreted	PTX3 expression was correlated with cell death caused by oxidative stress
Wang et al. (2016)	Mouse retina	4-HNE	RPE/inner BrM	In a mouse model of AMD, PTX3 was found to co-localize with factor H and control complement activation
Yamada et al. (2008)	Human retina	—	BrM/choriocapillaris	PTX3 immunohistochemical staining was documented in tissues obtained from AMD donors
Swinkels et al. (2018)	Human retina	—	BrM/choriocapillaris	PTX3 immunofluorescence staining was reported in tissues obtained both from AMD and non-AMD subjects, suggesting a role in retina homeostasis
Stravalaci et al. (2020)	Human vitreous	—	secreted	PTX3 was detected and quantitated in the humor vitreous of AMD and non-AMD donors, suggesting that other cell types of the retina (in addition to the RPE) can make the protein
Yang et al. (2014), Zhou and Hu. (2016), Erdenen et al. (2018), Elbana et al. (2019)	Human plasma/serum	—	circulating	Plasma/serum PTX3 levels were associated with DR
Chodkowski et al. (2018), Hokazono et al. (2018), Güngel et al. (2021)	Human plasma	—	circulating	No differences were found in the plasma levels of PTX3 measured in DR patients and diabetics without retinopathy
Mutlu et al. (2017)	Aqueous humor	—	secreted	PTX3 levels in the aqueous humor were associated with DR

a TNF-α, tumor necrosis factor-α; IL-1β, interleukin-1β; ox-LDL, oxidized low-density lipoprotein; 4-HNE, 4-Hydroxynonenal; NaIO_3_, sodium iodate.

## Structure/Function of the Long Pentraxin PTX3

Originally cloned in the early 1990s, PTX3 is the prototype of long pentraxins, which, along with the short pentraxins C reactive protein (CRP) and Serum Amyloid P component (SAP), make up a superfamily of evolutionary conserved proteins with distinctive structures and functions [reviewed in (Daigo et al., 2016)]. As opposed to CRP and SAP, whose synthesis is primarily induced in the liver by IL-6, PTX3 is expressed by a number of immune and non-immune cells (including RPE, endothelial and myeloid cells) at sites of inflammation and infection upon stimulation with inflammatory cytokines, microbial moieties, and intact microorganisms (Doni et al., 2019). Of relevance to AMD and DR, oxidative conditions [e.g., oxidized low density lipoproteins, ox-LDL (Norata et al., 2008; Yamada et al., 2008)], in addition to inflammatory cytokines (Breviario et al., 1992; An et al., 2008; Woo et al., 2013; Juel et al., 2015; Stravalaci et al., 2020), induce the synthesis of PTX3 both in RPE and endothelial cells (see below). Furthermore, expression of the human protein is controlled both in physiological and pathological conditions by epigenetic mechanisms (Rubino et al., 2017) and genetic polymorphisms (Garlanda et al., 2018).

Like other long pentraxins, the human PTX3 protomer is a multidomain glycoprotein (Inforzato et al., 2006) with a C-terminal pentraxin domain and an N-terminal region that fold into homo-octamers stabilized by noncovalent (coiled coils) and covalent (disulfide bonds) interactions (Inforzato et al., 2008; Inforzato et al., 2010). This structural organization mediates the protein’s interactions with a broad spectrum of ligands, which results into diverse functions in innate immunity (Porte et al., 2019), inflammation (Bottazzi et al., 2016), vascular biology (Presta et al., 2018; Ristagno et al., 2019) and tissue remodeling (Doni et al., 2016). In particular, PTX3 is a ligand of key complement activators [i.e., C1q (Nauta et al., 2003; Bally et al., 2019), ficolin-1 (Ma et al., 2013), ficolin-2 (Ma et al., 2009), mannose binding lectin, MBL (Ma et al., 2011), and C3b (Stravalaci et al., 2020)] and inhibitors [i.e., factor H (Deban et al., 2008), and C4 binding protein, C4BP (Braunschweig and Józsi, 2011)], and therefore modulates all three complement pathways. Also, PTX3 regulates the extravasation of leukocytes at sites of inflammation *via* its interaction with P-selectin, thus controlling the inflammatory response *via* complement-independent mechanisms (Deban et al., 2010). Interestingly, PTX3 binds selected fibroblast growth factors (FGFs), including FGF2 and FGF8b, and inhibits FGF-dependent angiogenic responses (Rusnati et al., 2004; Camozzi et al., 2006). Finally, this pentraxin is a key component of the hyaluronic acid-rich extracellular matrix (ECM) that forms in inflammatory and inflammation-like conditions (Scarchilli et al., 2007; Baranova et al., 2014). These properties, particularly the engagement of factor H and FGF2, might be relevant in the pathogenesis of AMD and DR, as discussed below.

## PTX3 as an Endogenous Rheostat of Complement Activation in AMD

Initial evidence of an involvement of PTX3 in the pathogenesis of AMD dates back to 2008, when Yamada et al. documented the presence of this protein in the macula of an 81-year-old male with early AMD by means of immunohistochemistry on post-mortem human eye specimens (Yamada et al., 2008). Using fluorescence microscopy techniques, we have afterward recapitulated these findings in an independent cohorts of AMD donors, and demonstrated that PTX3 is expressed in the eye of non-AMD donors too, where it localizes at the interface between the RPE and choroid, particularly in the intercapillary septa of the choriocapillaris (Swinkels et al., 2018). Also, we found the protein in the humor vitreous of both AMD and non-AMD donors (Stravalaci et al., 2020), suggesting that PTX3 is constitutively expressed in the human eye, where it might contribute to tissue homeostasis both in physiological and pathological conditions. Current literature indicates that PTX3 is locally made by the RPE in the presence of pro-inflammatory cytokines, such as TNF-α or IL-1β (An et al., 2008; Woo et al., 2013; Juel et al., 2015; Stravalaci et al., 2020), peroxidized lipids (i.e., 4-hydroxinonenanl, 4-HNE) (Wang et al., 2016), and ox-LDL (Hwang et al., 2019). Based on the notion that human leukocytes express PTX3 (Doni et al., 2019), it is plausible that eye-resident phagocytes (in addition to the RPE) might make the protein, including retinal microglia and Müller cells. Regardless of the cellular sources of PTX3 in the eye, this organ marginally contributes to the protein’s plasmatic pool, which therefore cannot predict the AMD status (Juel et al., 2015). Conceivably, it is the locally made protein (i.e., in the posterior segment of the eye) that contributes to AMD pathogenesis. So far, no large study has been conducted to assess associations between the ocular levels of PTX3 and AMD, however, in small cohorts of donors; trends of increasing protein concentration in the vitreous (Stravalaci et al., 2020) and staining intensity in the choriocapillaris (Swinkels et al., 2018) have been documented in AMD subjects. Also, it is interesting to notice that transcription of the *PTX3* gene in the human RPE/choroid region increases with age, although in an AMD-independent fashion (Juel et al., 2015).

The role of PTX3 in AMD has been investigated in diverse experimental settings. Hwang et al. reported that in the presence of sodium iodate (that induces oxidative stress), primary human H-RPE and ARPE-19 cells cultured *in vitro* increased PTX3 expression, and the newly synthesized protein impaired the transcription of antioxidant enzymes, while inducing that of AMD-associated genes (Hwang et al., 2019). These findings might suggest that PTX3 contributes to AMD development by accelerating RPE cell death, however a non-physiological chemical stimulus (i.e., sodium iodate) was used throughout the study, and the applied experimental setting did not consider the effect of PTX3 on activation of the complement system [a primary pathogenetic mechanism of AMD (Clark and Bishop, 2018)]. In this regard, PTX3 is a well-known ligand of complement factor H (CFH) (Deban et al., 2008), and genetic variations in the *CFH* gene, particularly the Y402H polymorphism in the complement control protein (CCP) module seven of the protein, are strongly associated with the risk of developing AMD, as anticipated above (Parente et al., 2017). PTX3 binds factor H at CCP7 and CCPs19-20, *via* its C- and N-terminal domains, respectively (Deban et al., 2008). Therefore, assembly and control of the factor H/PTX3 complex might be of functional relevance in retinal physiology and pathology. In this regard, in an animal model of AMD, genetic deficiency of PTX3 amplified complement activation induced by 4-HNE (a product of lipid peroxidation found in the AMD eye), with increased C3a levels and inflammasome activation, leading to IL-1β production by the RPE, and enhanced accumulation of macrophages in the choroid (Wang et al., 2016). These findings indicate that PTX3 mediates retinal homeostasis *in vivo*, especially in inflammatory conditions, whereby it promotes the recruitment of factor H and tames complement overactivation. Consistent with this view, PTX3 has been shown to co-localize with factor H in the murine inner BrM and RPE, where it controls factor H distribution and protects the RPE from complement dysregulation and inflammasome activation (Wang et al., 2016).

We have recently reported that PTX3 binds RPE cells in physiological conditions *in vitro*, however this interaction is impaired when these cells are stimulated with IL-1β and, to a lesser extent, TNF-α (to mimic the inflammatory microenvironment of AMD) (Stravalaci et al., 2020). Therefore, PTX3 cannot restrain complement on the RPE surface during inflammation, when expression of the AP-activating genes (*C3* and factor B, *FB*, but not *CFH*) is upregulated (Stravalaci et al., 2020). However, we have demonstrated that PTX3 recruits both factor H and C3b onto non-cellular surfaces (simulating the basement membrane of RPE and choroid, and the BrM), where it forms a stable ternary complex that acts as a “molecular brake” for complement activation (Stravalaci et al., 2020). This mechanism is likely relevant in the presence of the AMD-associated 402H variant of factor H, which has a more restricted specificity for sulfated GAGs compared to 402Y, and likely has decreased ability to control complement activation at ECM sites, such as the BrM (Clark and Bishop, 2018). Also, we have reported that PTX3 interacts with FHL-1 (in addition to factor H), and the Y402H polymorphism (that is retained in FHL-1) affects the binding of FHL-1 (but not factor H) to PTX3 (Swinkels et al., 2018). Immunolocalization studies indicate that FHL-1 is the major complement inhibitor in the BrM and intercapillary septa of the choriocapillaris, and passively diffuses through the BrM, whereas factor H cannot (Clark et al., 2014). Therefore, PTX3 might act as an ECM anchoring site for FHL-1 (in addition to factor H), and a “hot spot” for complement inhibition in the eye. Furthermore, the interaction of PTX3 with factor H has been proposed to promote complement-mediated phagocytosis [including clearance of apoptotic debris (Deban et al., 2008)], suggesting that this long pentraxin might take part in the RPE-dependent turnover of photoreceptor outer segments (POS), a fundamental process of retinal physiology (Kwon and Freeman, 2020). Overall, available *in vitro* and *in vivo* evidence points to a protective (rather than pathological) role of PTX3 in response to complement dysregulation in AMD.

## Emerging Roles of PTX3 in DR

Diabetes is underpinned by sterile, chronic, low-grade inflammation characterized by mildly elevated circulating levels of IL-1β (Donath and Shoelson, 2011). In line with this view, anti-inflammatory drugs, such as interleukin one receptor antagonist (Larsen et al., 2007), anti-IL-1β antibody (Cavelti-Weder et al., 2012), and salsalate (Goldfine et al., 2013) have been shown to lower hyperglycemia in type 2 diabetes patients. Most diabetic complications, including retinal diseases, are associated with endotheliopathy (endothelial dysfunction), which points to inflammatory vascular pathology as a major point of attention in the clinical management of diabetes (Rask-Madsen and King, 2013). Interestingly, PTX3 is produced by endothelial cells during inflammation (Breviario et al., 1992), and has been consistently proposed as a biomarker of vascular inflammation (Ristagno et al., 2019). This has prompted investigations into the role of PTX3 as a marker of disease in DR, with conflicting outcomes. In fact, higher PTX3 levels have been documented in the plasma (or serum) of DR patients, compared to that of diabetics with no retinopathy or non-diabetic volunteers (Yang et al., 2014; Zhou and Hu, 2016; Erdenen et al., 2018; Elbana et al., 2019), however these findings have not been recapitulated in other studies (Chodkowski et al., 2018; Hokazono et al., 2018; Güngel et al., 2021). Such lack of consistency is likely due to high variability in the plasmatic concentration of PTX3 in diabetic patients, as a reflection of varying extents of hyperglycemia-dependent damage to tissues and vascular beds. This makes it problematic to detect differences across small cohorts of patients (like those investigated so far). We propose that PTX3 is involved in the local rather than systemic inflammatory reaction to hyperglycemia. Our view is supported by a study reporting higher levels of the protein in the aqueous humor of patients with DR than in that of diabetics with no retinopathy or non-diabetic volunteers (Mutlu et al., 2017). This suggests that the diabetic damage to the retinal endothelium is perhaps more evident in adjacent tissues (like the vitreous) than is in the blood, where any PTX3 contribution from the retinal tissue is likely to be highly diluted. In line with this, no correlation has been found between serum hemoglobin A1c (HbA1c) and vitreous PTX3 levels (Mutlu et al., 2017).

PTX3 is produced by myeloid and endothelial cells in response to inflammatory stimuli, including TNF-α or IL-1β (Doni et al., 2019). The inner retina is a complex tissue that comprises various cells able to synthesize and release PTX3, and the diabetic microenvironment provides relevant pro-inflammatory triggers. DR is associated with endothelial dysfunction, microglia activation, and neurodegeneration (Stitt et al., 2016). In this context, microglia, endothelial cells, and neural cells are tissue-resident candidate producers of PTX3 within the retina. Retinal endothelial cells are particularly sensitive to damage by hyperglycemia. Indeed, early glycemia control is a primary therapeutic goal to avoid the development of DR complications (Yau et al., 2012). Poor control of glycemia and longer duration of diabetes are associated with loss of pericytes, thickening of the basement membrane, and pathological neovascularization in the vitreoretinal interphase, leading to proliferative DR. Since PTX3 binds to FGF2 (Camozzi et al., 2006), and inhibits its proangiogenic functions (Rusnati et al., 2004; Presta et al., 2018), there is potential for this pentraxin to provide an alternative to current anti-VEGF therapies for treatment of DR (and wet AMD), especially in non-responsive cases. Although inhibition of pathological preretinal neovascularization is the goal in the clinical management of advanced proliferative DR, it is important to underscore that in early-stage DR, intraretinal reparative angiogenesis is a desired biological outcome (Sapieha, 2012). Therefore, PTX3-dependent inhibition of angiogenesis (e.g., *via* binding and sequestration of FGF2) in the ischemic DR may drive disease progression (rather than regression) to proliferative retinopathy.

As outlined above, inflammation plays a major role in DR. In this regard, microglia are resident cells of the retina that modulate tissue inflammation. In physiological conditions, retinal microglia patrol the inner retina and maintain tissue homeostasis. However, in conditions of prolonged hyperglycemia and hypoxic stress, an activated microglial status is induced that promotes inflammation, including complement activation, and disease progression (Fumagalli et al., 2015). In this context, PTX3 might exert complement-modulating in addition to angiogenesis-inhibiting properties, with a more complex role in DR-associated retinal inflammation.

## Concluding Remarks

Clinical and pre-clinical evidence corroborates the inflammatory nature of AMD (Parmeggiani et al., 2013) and DR (Semeraro et al., 2019), and new molecules and processes are proposed that take part in their pathogenesis (Forrester et al., 2020). The inflammatory mediator PTX3 is emerging as a novel player in neurodegenerative disorders of the retina (summarized in Table 1). Endowed with modulatory properties towards complement (Haapasalo and Meri, 2019) and angiogenesis (Presta et al., 2018), this pentraxin is ideally positioned at the interface between immune and metabolic inflammation. In this regard, *in vivo* and *in vitro* data suggest that PTX3 acts as an endogenous inhibitor of complement overactivation and neovascularization in the human eye, thus holding promise as a pharmacological target for the treatment of AMD (Wang et al., 2016; Stravalaci et al., 2020). Available information points to this pentraxin as a biomarker and, possibly, a pathogenetic player of DR too, however more research is needed to address these hypotheses, with major regard to animal modelling of the disease and clinical investigations. Human data, in particular, are fragmented, likely due to small size of the studies so far conducted. Larger cohorts of patients are needed to overcome this limitation that integrate biochemical (e.g., protein concentration) and genetic (e.g., polymorphisms) information, perhaps accounting for epistatic interactions between this pentraxin and other pathogenetic drivers of DR (and AMD), including FGFs (and complement proteins).
